# Doxorubicin-poly (ethylene glycol)-alendronate self-assembled micelles for targeted therapy of bone metastatic cancer

**DOI:** 10.1038/srep14614

**Published:** 2015-09-30

**Authors:** Wei-liang Ye, Yi-pu Zhao, Huai-qiu Li, Ren Na, Fei Li, Qi-bing Mei, Ming-gao Zhao, Si-yuan Zhou

**Affiliations:** 1 Department of Pharmaceutics, School of Pharmacy, Fourth Military Medical University, Xi’an, 710032, China; 2 West Changle Sanatorium for Xi’an Army Retired Cadres of Fourth Military Medical University, Xi’an, 710032, China; 3 Department of Pharmacology, School of Pharmacy, Fourth Military Medical University, Xi’an, 710032, China

## Abstract

In order to increase the therapeutic effect of doxorubicin (DOX) on bone metastases, a multifunctional micelle was developed by combining pH-sensitive characteristics with bone active targeting capacity. The DOX loaded micelle was self-assembled by using doxorubicin-poly (ethylene glycol)-alendronate (DOX-hyd-PEG-ALN) as an amphiphilic material. The size and drug loading of DOX loaded DOX-hyd-PEG-ALN micelle was 114 nm and 24.3%. In pH 5.0 phosphate buffer solution (PBS), the micelle released DOX significantly faster than in pH 7.4 PBS. In addition, with the increase of incubation time, more red DOX fluorescence was observed in tumor cells and trafficked from cytoplasm to nucleus. The IC_50_ of DOX loaded DOX-hyd-PEG-ALN micelle on A549 cells was obviously lower than that of free DOX in 48 h. Furthermore, the *in vivo* image experimental results indicated that a larger amount of DOX was accumulated in the bone metastatic tumor tissue after DOX loaded DOX-hyd-PEG-ALN micelle was intravenously administered, which was confirmed by histological analysis. Finally, DOX loaded DOX-hyd-PEG-ALN micelle effectively delayed the tumor growth, decreased the bone loss and reduced the cardiac toxicity in tumor-bearing nude mice as compared with free DOX. In conclusion, DOX loaded DOX-hyd-PEG-ALN micelle had potential in treating bone metastatic tumor.

Some kinds of human cancers such as lung cancer, prostate cancer and breast cancer almost invariably metastasize to bone in their advanced stage. This is because the physiological microenvironment of bone is very suitable for tumor cells to adhere and proliferate^1^. The bone metastases can cause life threatening hypercalcemia, pathological fractures, spinal cord compression and chronic bone pain, and significantly lower the life quality of patients^2^.

The methods to treat bone metastases are radiotherapy, surgery and chemotherapy. However, their efficacies are limited due to the low selectivity to the multiple bone metastatic nodules and poor permeability in the bone tumor tissues^3^,^4^. Brubaker *et al.* found that docetaxel did not inhibit the growth of bone metastases tumor at the dose that was effective for subcutaneous implanted tumor^5^. This is probably because docetaxel is difficult to be delivered to the bone lesion sites because of the blood-bone barrier^6^.

Adult skeleton is composed of 50–70% mineral, 20–40% organic matrix, 5–10% water and 1–5% lipids. The main mineral component of bone is hydroxyapatite (HA). Bisphosphonates (BPs) show strong affinity with hydroxyapatite and can selectively accumulate in bone and bone metastases tumor^7^. After BPs is intravenously administered, approximately 50–75% of the injected dose binds to exposed bone mineral^8^. Additionally, an important characteristic of BPs is that the uptake of BPs in bone metastases tumor is 10 to 20-fold higher than in healthy bone tissue^9^. BPs can retain much of the binding affinity after they are connected with other molecules or drug carriers. Thus, BPs are used as ideal polymer/nanoparticle bone-targeting moiety. Alendronate (ALN), an amino bisphosphonate, is commonly used for the prevention and treatment of osteoporosis, bone metastases tumor and other bone related diseases. It is also a most commonly used bone-targeting ligand because it contains a primary amine and can readily be conjugated with carboxyl-containing drugs^10^,^11^,^12^. In order to increase the selectivity of chemotherapeutic drugs to bone metastases, some chemotherapeutic drugs were conjugated with BPs to treat bone metastases tumor. But their therapeutic efficacy was disappointed. For example, Keppler *et al.* found that the conjugation of cisplatin with BPs did not significantly improve the therapeutic efficacy of cisplatin in bone metastatic tumor model even at very high dose^13^. When doxorubicin (DOX) was conjugated with BPs by the amino bond, it showed no antitumor effect at all on bone metastases tumor^9^.

Polymer-drug conjugate is one of the most promising approaches for cancer therapy^14^,^15^. This is because the water-soluble polymers can increase the water-solubility of lipophilic drug, prolong the blood circulation time of drug and protect the drug from degradation. Eventually, the polymer-drug conjugate can accumulate at tumor site by the enhanced permeability and retention (EPR) effect, and thus enhance the therapeutic effect and decrease side effects. But the low drug loading and slow drug release rate limit the exertion of the efficacy of polymer-drug conjugates. For example, DBM2-PEG4000-S-PEG3000-GFLG-DOX conjugate was synthesized, and its drug loading was 3–4%. DBM2-PEG4000-S-PEG3000-GFLG-DOX showed low antitumor activity as compared with PEG5000-GFLG-DOX conjugate and HPMA copolymer-DOX conjugate because of the slow release rate of DOX from DBM2-PEG4000-S-PEG3000-GFLG-DOX^16^. Polymer-drug conjugate micelle is an important drug delivery system which can increase the drug loading with the characteristics of small size, extended release profile and better stability^17^,^18^. In our previous study, amphiphilic polymer (DOX-hyd-PEG-FA) was synthesized by conjugating the hydrophobic DOX with the hydrophilic poly (ethylene glycol) (PEG) through hydrazone bond. DOX-hyd-PEG-FA was used as micellar material to encapsulate free DOX to increase the drug loading. The segment of DOX provided a hydrophobic core to entrap free DOX, and the hydrophilic PEG shell reduced the uptake of micelle by mononuclear phagocytic system (MPS) and prolonged the blood circulation time of the micelle. The antitumor activity of DOX loaded DOX-hyd-PEG-FA micelle was greatly enhanced on tumor-bearing nude mice model^14^.

Because of the poor permeability, specific physiological and biochemical processes of bone tissue, it is difficult for drug to effectively accumulate in bone metastases tumor. Therefore, active bone-targeted drug delivery systems are needed. Hengst *et al.* used CHOL-TOE-BPs as the targeting moiety to modify liposome and the results suggested that the CHOL-TOE-BPs modified liposome was mainly localized at the bone site^19^. By using lysosomal enzyme (cathepsin B) responsive GFLG as a linker, N-(2-hydroxypropyl) methacrylamide copolymer-paclitaxel-alendronate (HPMA copolymer-PTX-ALN) was synthesized and used as a bone-targeting drug delivery system. In addition, HPMA copolymer-PTX-ALN nanoparticle showed the greatest antitumor efficacy on 4T1 mammary adenocarcinoma inoculated into the tibia as compared with PTX alone or in combination with ALN^20^. Furthermore, based on poly-(lactic-co-glycolide)-alendronate (PLGA-ALN), DOX-loaded bone-targeting nanoparticle was prepared. This drug delivery system was a promising strategy for active delivery drugs to bone tissues^21^,^22^. At the same time, Chen H *et al.* prepared DOX-loaded H40-star-PEG/ALE micelles. The affinity of H40-star-PEG/ALE micelle to bone was confirmed by the hydroxyapatite (HA) binding assay. The results indicated that the H40-star-PEG/ALE micelle was a promising bone-targeted drug carrier for bone metastases tumor^23^.

In this paper, in order to increase the therapeutic effect of DOX on bone metastases, doxorubicin-poly (ethylene glycol)-alendronate (DOX-hyd-PEG-ALN) copolymer was synthesized. The hydrophilic and hydrophobic segment was conjugated by the hydrazone bond which was responsive to the acidic microenvironment of endolysosome in tumor cell^24^. Then the copolymer self-assembled into micelle. The free DOX was encapsulated into the micelle (Fig. 1). The binding affinity of the DOX-hyd-PEG-ALN micelle with HA was evaluated. In addition, the pH sensitivity as well as drug release properties of DOX-hyd-PEG-ALN micelle was investigated. Finally, the *in vitro* cytotoxicity and *in vivo* anti-tumor activity of DOX loaded DOX-hyd-PEG-ALN micelle were further studied.

## Materials and Methods

### Materials

Alendronate (ALN), hydroxyapatite (HA), N-hydroxysuccinimide (NHS), 3-(4,5-dimethylthiazol-2-yl)-2,5-diphenyl tetrazolium bromide (MTT), 1-ethyl-(3-dimethylaminopropyl)-carbodiimide (EDCI) and trifluoroacetic acid (TFA) were the product of Sigma-Aldrich company (St. Louis, MO). α,ω-dicarboxyl poly(ethylene glycol) (HOOC-PEG-COOH, average molecular weight was 5000 Da) was obtained from Shanghai Yare Biotech Inc.(Shanghai, China). Doxorubicin·HCl (DOX·HCl) was the product of Hisun Pharmaceutical Co. (Zhejiang province, China). 4′,6-diamidino-2-phenylindole (DAPI) was the product of Invitrogen Technologies Company (Carlsbad, USA). All other analytical grade chemicals were purchased from J&K company (Beijing, China). A549 cell was purchased from the Shanghai Institute of Biochemistry and Cell Biology, Chinese Academy of Science. Primary antibodies rabbit anti-cyclin A and mouse anti-cyclin D1, and the secondary antibodies goat anti-mouse IgG and goat anti-rabbit IgG were purchased from Proteintech (USA).

Female athymic nude mice were supplied by the Experimental Animal Center, Fourth Military Medical University. All animal experimental procedures were executed in accordance with protocols that were authorized by the Animal Care and Use Committee, Fourth Military Medical University (approval date: 18/10/2014, number: 15317).

### Synthesis of DOX-hyd-PEG-ALN

**Figure 2:**
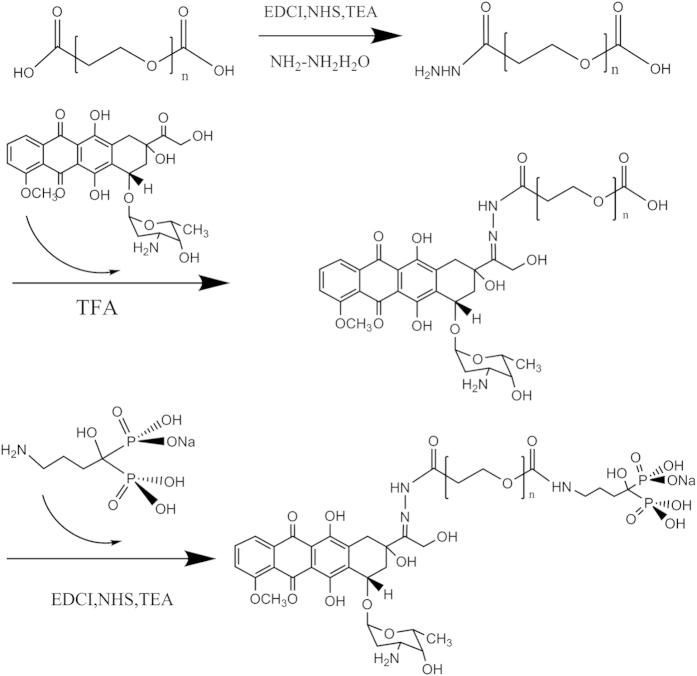
The synthetic route of DOX-hyd-PEG-ALN conjugate.

The DOX-hyd-PEG-COOH was synthesized according to our previously reported study^14^, and the synthetic route is showed in Fig. 2. Firstly, HOOC-PEG-COOH was activated by EDCI and NHS in dimethylsulfoxide (DMSO). Then NH_2_NH_2_·H_2_O was added into the solution and the reaction mixture was stirred for 24 h to obtain HOOC-PEG-CONHNH_2_. Finally, 80 mg HOOC-PEG-CONHNH_2_ was dissolved in 20 mL DMSO, and then 20 mg DOX and 10 μL trifluoroacetic (TFA) were added into the solution and stirred for more 48 h in darkness. The reaction mixture was completely dialyzed in deionized water and lyophilized to get DOX-hyd-PEG-COOH conjugate.

ALN was conjugated with DOX-hyd-PEG-COOH by amino bond^25^. In brief, 90 mg DOX-hyd-PEG-COOH was activated by 43 mg NHS, 78 mg EDCI and 20 μL TEA in 15 mL dioxane in darkness at room temperature for 8 h. Then 120 mg ALN was dissolved in 15 mL H_2_O and dropped into the above reaction mixture. During the reaction process, the pH value of reaction mixture was maintained at 7 by dropwisely adding 0.2 mol/L NaOH solution. After 2 h, the pH value of reaction mixture was quickly adjusted to 9 to stop the reaction by using 0.2 mol/L NaOH solution. Finally, the reaction mixture was dialyzed in deionized water and lyophilized to obtain DOX-hyd-PEG-ALN conjugate. DOX-hyd-PEG was synthesized as a control conjugate by using methoxy-PEG-COOH to replace HOOC-PEG-COOH.

### Preparation and characterization of the micelle

The micelle was prepared by the dialysis method^26^. In brief, 20 mg DOX-hyd-PEG-ALN polymer, 4 mg DOX and 4 μL TEA were dissolved into 20 mL DMSO. The solution was stirred at 80 °C for 24 h and then dialyzed against deionized water for 72 h in order to remove the unloaded drug and obtain the DOX-loaded DOX-hyd-PEG-ALN micelle. DOX-loaded DOX-hyd-PEG micelle was prepared with the same method.

The micelle size, poly dispersity index and zeta potential were determined by using Beckman Coulter Particle Analyzer (Fullerton, California, USA). All measurements were performed at room temperature and the data were obtained from the average of three measurements. The morphology of micelle was examined by using transmission electron microscopy (TEM, JEOL-100CXII, Japan)^27^. The critical micelle concentration (CMC) of the micelle was determined by fluorescent spectroscopic method with pyrene as fluorescence probe^28^. The micelle was dispersed in phosphate buffer solution (PBS, pH 7.4) containing 20% fetal bovine serum. Its size and PDI (polydispersity index) at room temperature were consecutively recorded for five days.

### Determination of drug loading and entrapment efficiency of micelle

To determine the drug loading and entrapment efficiency, the freeze-dried micelles were dispersed in 4 mL of 1 mol/L HCl and shook in water bath at 50 °C for 6 h to produce DOX^28^. The DOX concentration was measured by using fluorescence spectroscopy (970 CRT Spectrofluorophotometer, Shanghai Precision and Scientific Instrument Co. Ltd, Shanghai, China). The measurement was performed in triplicate. Drug loading and entrapment efficiency was respectively calculated by the following equations:
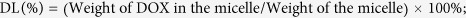

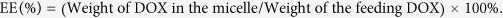


### pH-responsive characteristics of the micelle

To investigate the pH-responsive characteristic of the DOX-loaded DOX-hyd-PEG-ALN micelle, the size change was evaluated by DLS^29^. Briefly, 2.5 mg DOX loaded DOX-hyd-PEG-ALN micelle was dispersed in 5 mL phosphate buffer solution (pH 5.0 and pH 7.4). The size was analyzed by using Beckman Coulter Particle Analyzer (Fullerton, California, USA) at 0 h and 4 h respectively after DOX loaded DOX-hyd-PEG-ALN micelle was dispersed. The morphology change was also observed by TEM.

The release of DOX from DOX loaded DOX-hyd-PEG-ALN micelle was investigated by using dialysis method^30^. In brief, 20 mg of the micelle was dispersed in 5 mL PBS (pH 5.0, 6.5 and 7.4) and kept in dialysis bag (MWCO: 1000Da). The dialysis bag was then immerged in 95 mL PBS (pH 5.0, 6.5 and 7.4) and shook in water bath at 37 °C. 3 mL of release medium was collected at various time intervals for analysis and replaced with the same amount of fresh PBS. The released DOX in PBS was analyzed by using fluorescence spectroscopy as aforementioned method. The measurement was conducted in triplicate.

### HA binding kinetics

The binding kinetics of the DOX, DOX loaded DOX-hyd-PEG micelle and DOX loaded DOX-hyd-PEG-ALN micelle with HA was assessed according to a previously reported procedure^31^,^32^. Briefly, in a falcon tube, 1 mg of DOX, DOX loaded DOX-hyd-PEG micelle or DOX loaded DOX-hyd-PEG-ALN micelle was dispersed in 10 mL PBS. Then, 100 mg HA was added into the tube and gently shaken at room temperature. At the same time, DOX, DOX loaded DOX-hyd-PEG micelle and DOX loaded DOX-hyd-PEG-ALN micelle were respectively incubated at the same condition without HA. After 5 min, 15 min, 30 min, 45 min and 60 min, the mixture solution was centrifuged (4000 rpm, 5 min) and the absorbance of the supernatant (diluted with the same volume of DMSO) was measured by UV-VIS spectrophotometer at 233 nm (Beckman DU-800 spectrophotometer, USA). The binding percentage with HA was calculated according to the following formula:
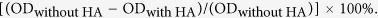


### Cell culture condition

Human lung cancer cell line (A549), which is easy to metastasize to bone^33^, was obtained from Shanghai Institutes of Biology Sciences, Chinese Academy of Sciences (Shanghai, China). A549 cells were cultured in a RPMI 1640 medium containing 10% fetal bovine serum, 100 units/mL streptomycin and 100 units/mL penicillin at 37 °C and 5% CO_2_ humidified atmosphere.

### Cellular uptake of micelle

The A549 cells were planted into coverslip-containing 24-well plate at a density of 1.5 × 10^5^ cells/mL and were cultured overnight at 37 °C. The cell culture medium then was replaced with fresh cell culture medium containing DOX loaded DOX-hyd-PEG-ALN micelle (equivalent DOX concentration: 10 μg/mL) and was incubated for 5 min, 30 min or 3 h at 37 °C. Then, the cells were slightly rinsed with PBS for three times and treated with 4′,6-diamidino-2-phenylindole (DAPI, 100 ng/mL) for 15 min to stain nucleus. Finally, the cells were slightly rinsed with PBS for three times and fixed with 4% paraformaldehyde. Cover slip was placed on glass slide and the uptake of DOX was observed by 80i fluorescence microscope (Nikon Corporation, Tokyo Japan). By using ImageJ software, the mean fluorescence intensity (MFI) of DOX in the nucleus or cytoplasm was calculated in an area of 4 μM^2^ for each sample.

Flow cytometry was also used to monitor the cellular uptake of micelle by A549 cells. Firstly, the A549 cells were planted into 6-well plates with a density of 2 × 10^6^ cells/mL and cultured with DOX loaded DOX-hyd-PEG-ALN micelle (equivalent DOX concentration: 10 μg/mL) for 5 min, 30 min and 3 h at 37 °C. The cells were then washed, re-suspended in 0.2 mL PBS, and quantitatively determined by flow cytometry (Becton Dickinson FACScan, USA).

### *In vitro* cytotoxicity assay

The toxicity of free DOX and DOX loaded DOX-hyd-PEG-ALN micelle on A549 cells was evaluated by using MTT method. The cells were planted into 96-well plates with a density of 6 × 10^3^ cells per well and cultured for 24 h. After the medium was removed from the well, and a series of concentration of free DOX or DOX loaded DOX-hyd-PEG-ALN micelle dissolved in fresh cell culture medium were added. The cells were cultured for 24 h or 48 h. The 5 mg/mL of MTT solution was added and the cells were incubated for more 4 h. After the culture medium was removed, DMSO (150 μL) was added. By using a CODA Automated EIA Analyzer (Bio-Rad Laboratories, Hercules, CA), the absorbance of DMSO solution in each well was determined at 570 nm.

### Cell cycle analysis

Cell cycle was analyzed according to the previous literature^34^. Firstly, A549 cells were planted in 6-well plates with a density of 2 × 10^6^ cells per well and cultured for overnight to allow cells attachment. The cell culture medium was then replaced with fresh cell culture medium that contained free DOX or DOX loaded DOX-hyd-PEG-ALN micelle (equivalent DOX concentration: 10 μg/mL), and the cells were cultured for 24 h or 48 h at 37 °C. The cells were harvested and then re-suspended in 70% ice cold ethanol and frozen overnight. After being centrifugated at 800 × g for 5 minutes, the cells were washed with cold PBS (pH 7.4) and resuspended in PBS containing PI (propidium iodide, 50 μg/mL), Triton X-100 (0.1%, v/v), and DNase-free RNase (1 μg/mL) for 1 h at 37 °C. Finally, the cells were analyzed by flow cytometry (Becton Dickinson FACScan, USA). For each sample, a total of 10,000 events were acquired. Based on cell size, cell debris and cellular aggregates were gated and excluded. In addition, the effect of free DOX or DOX loaded DOX-hyd-PEG-ALN micelle on cell cycle protein cyclin A and cyclin D1 was determined by western blot according to the literature^35^.

### Biodistribution of DOX *in vivo*

A549 cells (1 × 10^7^ cells/animal) were injected into tibia of the male nude mice (23 ± 2 g) to induce the bone metastases. When the tumor volume reached about 300 mm^3^, 200 μL of normal saline, free DOX (5 mg/kg) and DOX loaded DOX-hyd-PEG-ALN micelle (5 mg DOX/kg) were respectively administered to mice by tail vein injection. Twenty four hours after the administration of micelle, the major organs, such as heart, liver, spleen, kidney, lung and bone with tumor were removed and analyzed by using Caliper IVIS Lumina II *in vivo* image (Caliper Life Science, USA). The fluorescence intensity in organs and bone tumor tissues was quantitatively analyzed by using Living Image 4.2 software. To investigate the details of DOX distribution in tumor site, the tumor tissues were sectioned into 5 μm thickness and the nucleus was stained with DAPI. Then the tissue section was observed by using confocal laser scanning microscopy. The mean fluorescence intensity (MFI) of DOX in the nucleus or cytoplasm was calculated by using aforementioned method.

### *In vivo* antitumor activity

Five no-tumor male nude mice were used as normal control (no tumor). A549 cells (1 × 10^7^ cells/animal) were injected into the tibia of the male nude mice to induce the bone metastases. After 10 days of tumor cells inoculation, mice were randomized in three different groups with 5 in each group: (i) normal saline treated group, (ii) DOX (5 mg/kg) treated group, (iii) DOX loaded DOX-hyd-PEG-ALN micelle (5 mg DOX/kg) treated group. Treatment was carried out once in a week by tail vein injection for 35 days. During the treatment, the body weight of tumor-bearing mice was recorded as index of toxicity. The long diameter (L) and the short diameter (W) of a tumor were measured with a caliper every 3 days. The tumor volume was calculated by using the formula: volume = LW^2^/2. At the end of the experiment, the tumor and heart tissue of tumor-bearing mice were removed and stained by hematoxylin and eosin (H & E) to observe tissue injury.

Additionally, leg bones (femur, tibia and fibula) were dissected from the killed mice and stored in 4% paraformaldehyde solution. An *ex vivo* micro-computed tomography (micro-CT) analysis of mouse leg bones was performed to validate the bone injury. Siemens Inveon Micro-CT (Munich, Germany) was used. Experimental parameters were as follows: X-ray: 50 keV, 500 μA; resolution: 111.25 μm. Micro-CT leg bones 3D reconstruction was performed by the open-source software Osirix. 3D microstructural properties of leg bone including bone volume fraction (BV/TV) and bone mineral density (BMD) were calculated by using software of Siemens Inveon Micro-CT.

### Statistical analysis

Experiments were done in triplicates. The results are expressed as mean ± SD. Statistical analyses were performed with Graph Pad Prism.

## Results and Discussion

### Characterization of DOX-hyd-PEG-ALN

**Figure 3:**
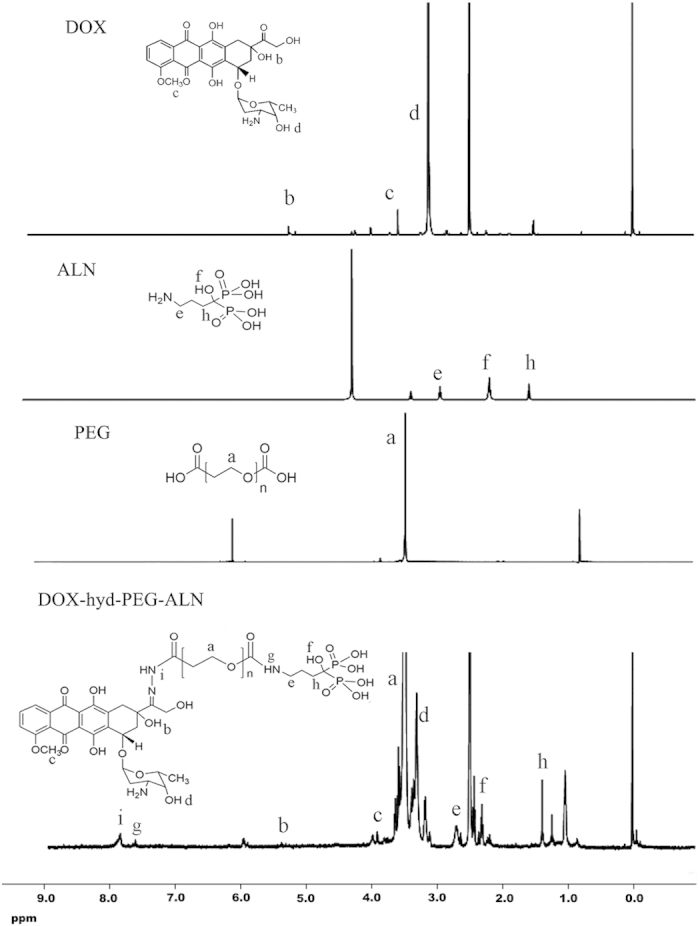
^1^H NMR spectrum of DOX, ALN, PEG and DOX-hyd-PEG-ALN.

**Figure 4:**
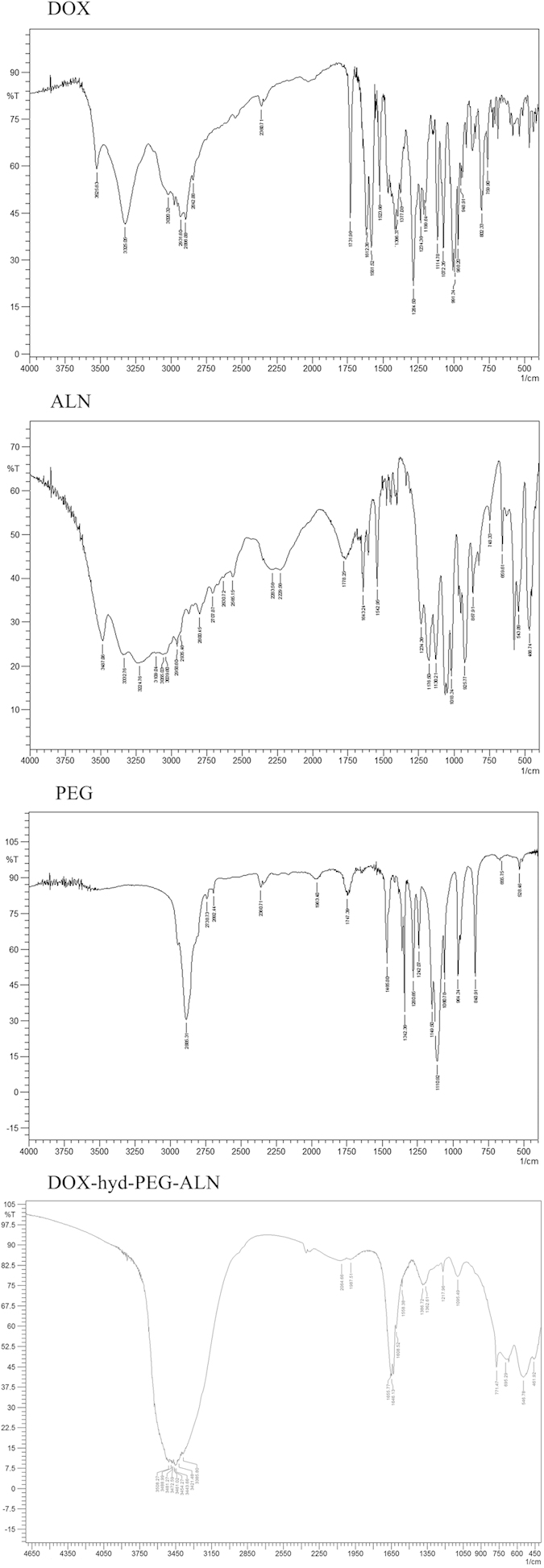
IR spectrum of DOX, ALN, PEG and DOX-hyd-PEG-ALN.

The ^1^H NMR spectrum of DOX-hyd-PEG-ALN is showed in Fig. 3. The ALN in DOX-hyd-PEG-ALN was verified by the signals at peak e, f and h (δ = 2.7, 2.3 and 1.4 ppm, respective). Moreover, the DOX in DOX-hyd-PEG-ALN was verified by the signals at peak b, c and d (δ = 5.4, 4.0 and 3.3 ppm, respective)^24^. The appearance of signal at peak i (δ = 7.8 ppm, −N=NH−) confirmed the hydrazone bond connection between DOX and PEG. The appearance of signal at peak g (δ = 7.6 ppm, −CONH−) confirmed the amide bond connection between ALN and PEG. The PEG backbone in the conjugate was verified by the signal at peak a (δ = 3.6 ppm)^14^. The IR spectrum of the DOX-hyd-PEG-ALN is showed in Fig. 4. The peak at 3300 ~ 3500 was corresponding to amino bond stretching bands, peak at 1634 was corresponding to the C=C stretching vibration band, peak at 1608 was corresponding to the hydrazone bond stretching vibration band, peaks at 461 and 546 were corresponding to the O−P−O stretching bands, and peak at 1362 was corresponding to the P=O vibration band^36^,^37^.

### Characterization of micelle

**Figure 5:**
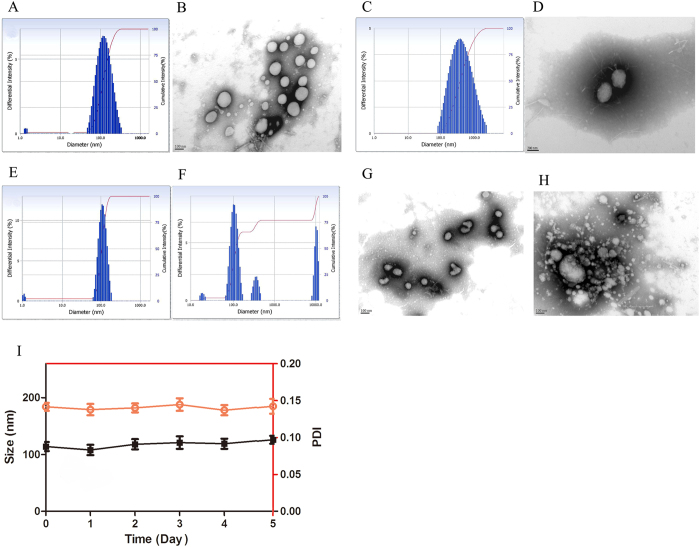
The size distribution (A) and TEM image (B) of DOX-loaded DOX-hyd-PEG-ALN micelle. The size distribution (**C**) and TEM image (**D**) of DOX loaded DOX-hyd-PEG micelle. The pH responsive characteristics of DOX loaded DOX-hyd-PEG-ALN micelle monitored by DLS and TEM in pH 7.4 medium for 4 h (**E,G**) and in pH 5.0 medium for 4 h (**F,H**). n = 3. Stability of DOX loaded DOX-hyd-PEG-ALN micelle in the presence of 20% fetal bovine serum (FBS) in PBS at room temperature (**I**). n = 3.

The particle size and size distribution of the DOX loaded DOX-hyd-PEG-ALN micelle and DOX loaded DOX-hyd-PEG micelle are showed in Table 1, Fig. 5A,C. The average size of the DOX loaded DOX-hyd-PEG-ALN micelle and DOX loaded DOX-hyd-PEG micelle was approximately 114 nm and 278 nm, respectively. The size of DOX loaded DOX-hyd-PEG-ALN micelle was smaller than that of DOX loaded DOX-hyd-PEG micelle. This was probably because that ALN changed the physiochemical properties of surface of DOX loaded DOX-hyd-PEG-ALN micelle (for example the absolute value of zeta potential of DOX loaded DOX-hyd-PEG-ALN micelle was greater than that of DOX loaded DOX-hyd-PEG micelle), which reduced the aggregation of the micelle^38^. The result was consistent with what was reported in the literature. For example, it was reported that the size of cholic acid modified nanoparticles CA-PLA-TPGS (112.9 ± 3.1 nm) was smaller than PLA-TPGS nanoparticles (125.7 ± 3.5 nm)^39^. Beside, the size of DOX-loaded M-PLGA-b-TPGS NPs (110.9 nm) was much smaller than DOX-loaded PLGA NPs (143.7  nm)^40^. The size of the DOX loaded DOX-hyd-PEG-ALN micelle was in the suitable size range for accumulating in tumor tissue by EPR effects^41^. In addition, PDI is a very important index of size distribution^42^,^43^. Zahra Hami *et al.* prepared DOX loaded DOX-Hyd-PLA-PEG-FOL micelle with DOX and PLA as hydrophobic core. The size and PDI of DOX-Hyd-PLA-PEG-FOL micelle were 182 nm and 0.28 ± 0.04, respectively^44^. A redox-responsive star-shaped PECLss-FA micelle was prepared. The size and PDI of star-shaped PECLss-FA micelle were 200 nm and 0.279, respectively^29^. Besides, Craparo EF *et al.* prepared beclomethasone dipropionate loaded PHEA-PEG2000-DSPE micelles. The size and PDI of this micelle were 200 nm and 0.4, respectively^45^. Recently, FA-PECL-SS-CPT was synthesized by using disulfide as a linker between poly(ethylene glycol)-b-poly(ε-caprolactone) (PECL) and camptothecin (CPT). With PCL and CPT as hydrophobic core and PEG as hydrophilic corona, FA-PECL-SS-CPT assembled itself into micelle. The size and PDI of FA-PECL-SS-CPT micelle were 155 nm and 0.512, respectively^46^. The size and PDI of DOX loaded DOX-hyd-PEG-ALN micelle was 114 nm and 0.142 ± 0.08, respectively. Thus, compared with above reported micelles, the size distribution of DOX loaded DOX-hyd-PEG-ALN micelle was relatively narrow. The TEM result showed that DOX loaded DOX-hyd-PEG-ALN micelle was generally spherical in shape (Fig. 5B).

Zeta potential is an important index of micelle. As displayed in Table 1, the zeta potential of the DOX loaded DOX-hyd-PEG micelles and DOX loaded DOX-hyd-PEG-ALN micelles was −16.6 mV and −19.3 mV, respectively. The negative surface charge of the micelle was due to the presence of ALN segment^47^. The CMC value of DOX loaded DOX-hyd-PEG-ALN micelle was 2.38 mg/L, which was low enough to ensure the stability of micelle in blood circulation and in body fluids^23^. Moreover, the DOX loaded DOX-hyd-PEG-ALN micelle showed high drug loading. This was because DOX was connected with PEG by hydrazone bond and was used as hydrobolic moiety in micellar material. In acidic environment, the hydrazone bond was broken, which resulted in the disaggregation of micelle and the release of conjugated DOX and encapsulated free DOX. Drug loading capacity is very important for drug delivery system. The drug loading capacity changed greatly in different drug delivery systems^48^. By using nanoprecipitation and single emulsion method, Swami A *et al.* prepared the bortezomib loaded ALN-PEG-PLGA nanoparticle to deliver bortezomib to bone. The drug loading was 0.04% and 0.74% respectively^49^. Clementi C *et al.* prepared the PTX-PEG-ALN micelle to delver paclitaxel (PTX) to bone to treat tumor bone metastases. The drug loading of PTX-PEG-ALN micelle was 4.7%^50^. In addition, based on hyperbranched Boltorn H40 and poly (ethylene glycol), H40-star-PEG/ALE polymer was synthesized and used to prepare bone targeting micelle to deliver DOX to bone. The drug-loading of H40-star-PEG/ALE micelle was 5%^23^. The drug loading of DOX loaded DOX-hyd-PEG-ALN micelle was (24.3 ± 3.7)%. Thus, the high drug loading was a very important merit of DOX loaded DOX-hyd-PEG-ALN micelle.

The adsorption capacity of nanoparticle with plasma opsonin is closely related to the uptake of nanoparticle by MPS^51^, which further decides the stability and fate of nanoparticle in blood circulation. Kastantin M *et al.* found that the micelle breakup rate was similar in plasma and bovine serum albumin (BSA) solutions. The adsorption with albumin was the main process involving in micelle breakup in blood^52^. Thus, in order to demonstrate the serum stability of DOX loaded DOX-hyd-PEG-ALN micelle, particle size was monitored in the presence of 20% fetal bovine serum (FBS) in PBS (pH = 7.4)^53^,^54^. The stability of the micelle in serum-containing PBS is showed in Fig. 5I. The results indicated that in the presence of 20% fetal bovine serum (FBS) in PBS (pH = 7.4), the DOX loaded DOX-hyd-PEG-ALN micelle was stable in 5 days. These results implied that micelle could maintain stability in the bloodstream.

### pH responsive behavior of micelle

In pH 7.4 PBS, the size of DOX loaded DOX-hyd-PEG-ALN micelle did not obviously change in 4 h (Fig. 5E). However, in pH 5.0 PBS, the size of DOX loaded DOX-hyd-PEG-ALN micelle significantly increased from 106 nm to 664 nm in 4 h (Fig. 5F). This was because the hydrazone bond in micelle was broken in pH 5.0 medium, the DOX loaded DOX-hyd-PEG-ALN micelle became looser, which resulted in the increase of size. In addition, in pH 7.4 medium, TEM image of DOX loaded DOX-hyd-PEG-ALN micelle was generally spherical in shape with good dispersibility (Fig. 5G). However, in pH 5.0 medium, the micelle was disassembled and was obviously aggregated (Fig. 5H).

### Drug release characteristics *in vitro*

**Figure 6:**
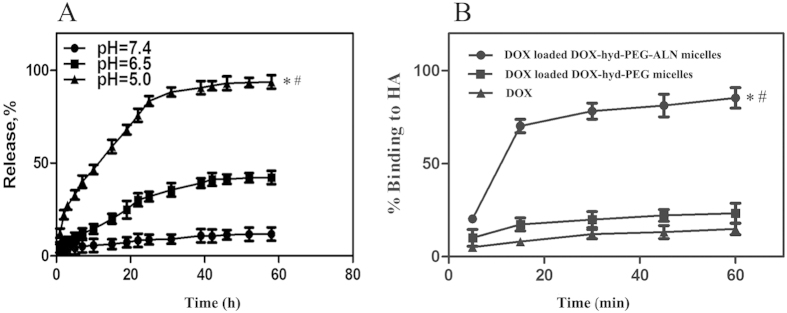
Accumulative release characteristics of DOX from DOX loaded DOX-hyd-PEG-ALN micelle at different pH medium (A). Data are presented as the average ± standard deviation (n = 3). *P < 0.05 vs pH7.4; ^#^P < 0.05 vs pH6.5. Binding kinetics of DOX, DOX loaded DOX-hyd-PEG micelle and DOX loaded DOX-hyd-PEG-ALN micelle with the HA (**B**). Data are presented as the average ± standard deviation (n = 3). *P < 0.05 vs DOX; ^#^P < 0.05 vs DOX-loaded DOX-hyd-PEG micelles.

The drug release behavior of the DOX loaded DOX-hyd-PEG-ALN micelle was investigated under a simulated physiological condition (pH 7.4) and an acidic microenvironment (pH 6.5 and 5.0) at 37 °C. As showed in Fig. 6A, the speed and amount of DOX released from the DOX loaded DOX-hyd-PEG-ALN micelle was strongly dependent on pH value. DOX loaded DOX-hyd-PEG-ALN micelle released DOX faster at pH 5.0 and 6.5 than at pH 7.4. The faster release of DOX from the DOX loaded DOX-hyd-PEG-ALN micelle in acidic environment was due to the acid-cleavable characteristics of the hydrazone bond in micellar material. It was expected that the DOX loaded DOX-hyd-PEG-ALN micelle was stable in blood circulation and accumulated in tumor tissue through EPR effect. Once in endolysosome of tumor cells, the DOX was released out from DOX loaded DOX-hyd-PEG-ALN micelle because of the acidic microenvironment of endolysosome, and DOX subsequently diffused into the cytoplasma of tumor cells and trafficked to the nucleus^55^.

### HA binding kinetics of micelle

For tumor metastasized bone tissue, the main constituent of skeleton HA was exposed in lysis sites. It is well-documented that ALN has a strong affinity with bone tissue because of its hydroxyl group and phosphonate group^56^. To estimate the binding capacity of DOX loaded DOX-hyd-PEG-ALN micelle with bone tissue, HA binding assay was carried out *in vitro*. The binding kinetic is showed in Fig. 6B. After 15 min of incubation, approximately 70% of DOX loaded DOX-hyd-PEG-ALN micelle was bound with HA and reached the plateau within 30 min. In contrast, compared with DOX loaded DOX-hyd-PEG-ALN micelle, the DOX loaded DOX-hyd-PEG micelle showed obviously lower adsorbability with HA. The above results implied that the DOX loaded DOX-hyd-PEG-ALN micelle could quickly target at the bone lysis sites.

### Cellular uptake of micelle

**Figure 7:**
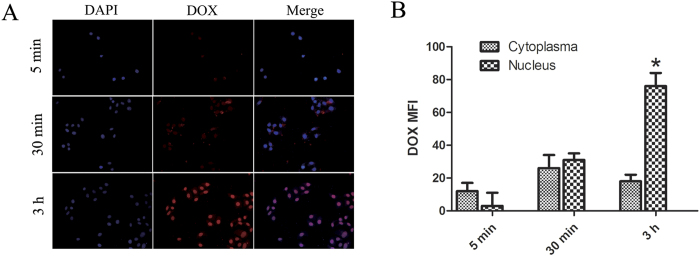
Fluorescence microscope images (A) of A549 cells incubated with DOX loaded DOX-hyd-PEG-ALN micelle for 5 min, 30 min and 3 h at 37 °C. Quantitative analysis of fluorescence microscope images (**B**), *P < 0.05 vs cytoplasma at the same time point. DOX concentration was 10 μg/mL. The pink region shows the localization of DOX (red) in the nucleus (blue).

In this experiment, DOX itself was used as a fluorescence marker^57^. As showed in Fig. 7A,B, there was little red DOX fluorescence in the cytoplasm and nucleus of the A549 cells when the cells were incubated with the DOX loaded DOX-hyd-PEG-ALN micelle for 5 min. The intracellular DOX fluorescence intensity gradually increased when the incubation time increased from 5 min to 30 min. After A549 cells were incubated with DOX loaded DOX-hyd-PEG-ALN micelle for 30 min, red DOX fluorescence intensity in the cytoplasm was the same as in the nucleus. After the A549 cells were incubated with DOX loaded DOX-hyd-PEG-ALN micelle for 3 h, the DOX red fluorescence almost localized completely in the nucleus of the A549 cells. This result indicated that DOX loaded DOX-hyd-PEG-ALN micelle was taken up efficiently by A549 cells, and DOX was released from DOX loaded DOX-hyd-PEG-ALN micelle and trafficked to the nucleus of A549 cell^58^.

**Figure 8:**
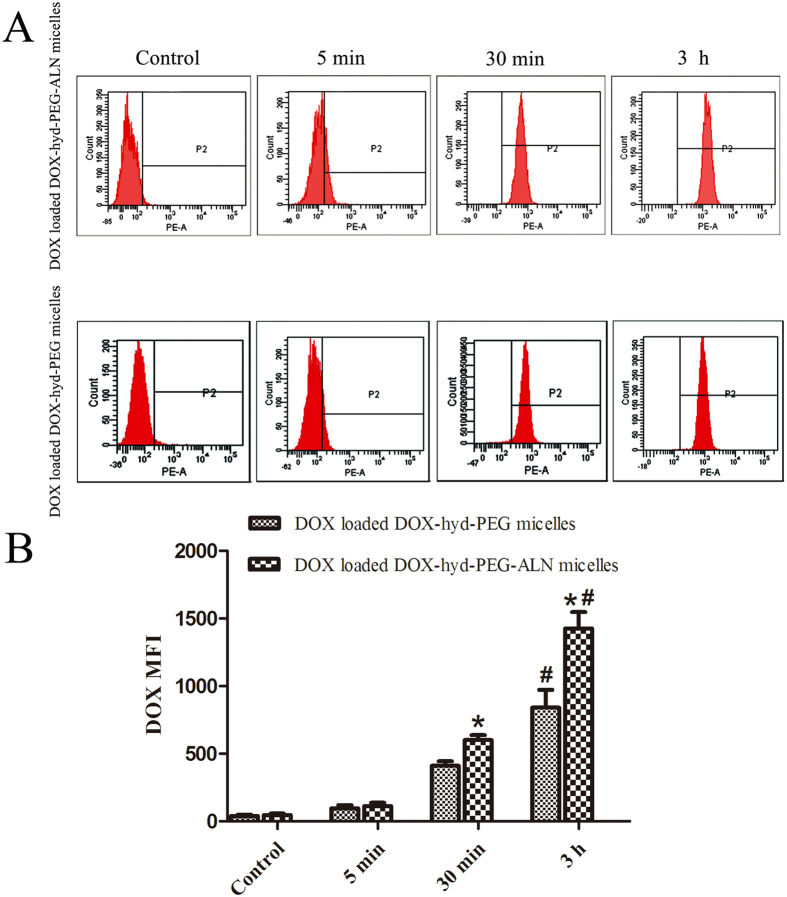
Flow cytometry images (A) of A549 cells incubated with DOX loaded DOX-hyd-PEG-ALN micelle and DOX loaded DOX-hyd-PEG micelle for 5 min, 30 min and 3 h at 37 °C. Quantitative analysis of flow cytometry images (**B**), *P < 0.05 vs DOX loaded DOX-hyd-PEG micelle at the same time point; ^#^P < 0.05 vs 30 min of the same micelle. DOX concentration was 10 μg/mL.

The cellular uptake of DOX loaded DOX-hyd-PEG-ALN micelle and DOX loaded DOX-hyd-PEG micelle by A549 cells were further evaluated by flow cytometry. The results are showed in Fig. 8A,B. The fluorescence intensity augmented with the increase of incubation time. The cellular uptakes of DOX loaded DOX-hyd-PEG-ALN micelle was greater than that of DOX loaded DOX-hyd-PEG micelle on A549 cells. The TEM results showed that both of DOX loaded DOX-hyd-PEG-ALN micelle and DOX loaded DOX-hyd-PEG micelle were generally spherical in shape. Thus, the high intracellular uptake efficiency of DOX loaded DOX-hyd-PEG-ALN micelle was attributed to its small size. Particle size is regarded as a key factor in the cellular uptake of polymeric particles. The cellular uptake and permeability increase with the decrease of particle size^59^,^60^. For example, Langston Suen WL *et al.* reported that the cellular uptake of 50 nm nanoparticle was faster than that of 250 nm particle^61^.

### Cytotoxicity of micelle

**Figure 9:**
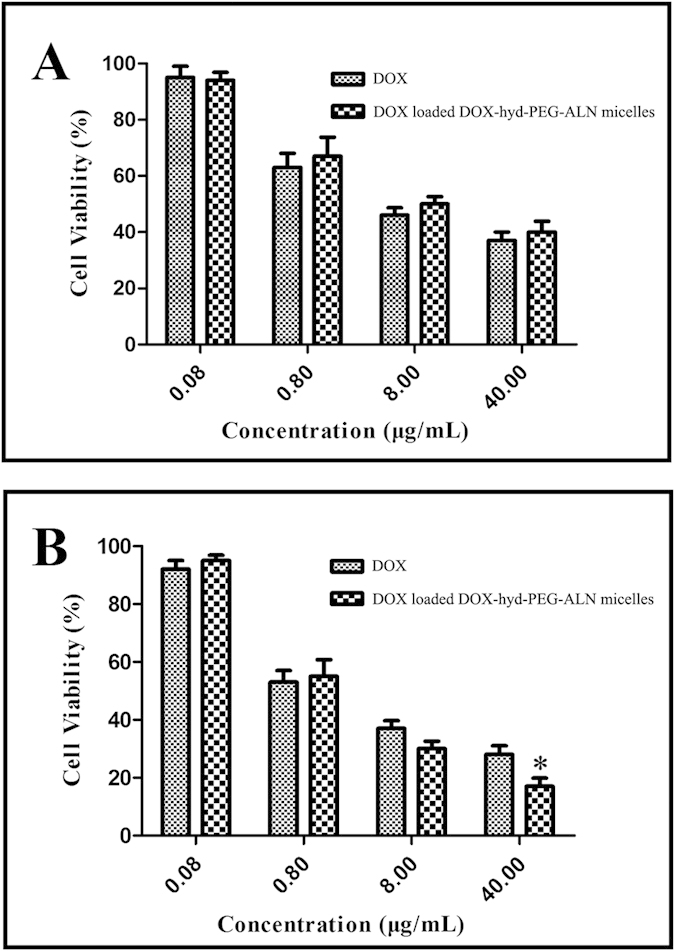
Cytotoxicity free DOX and DOX loaded DOX-hyd-PEG-ALN micelle on A549 cells in 24 h (A) and 48 h (B). Data are presented as the average ± standard deviation (n = 5). *p < 0.05 vs DOX at the same concentration of free DOX.

The cytotoxicity of DOX loaded DOX-hyd-PEG-ALN micelle on A549 cells is showed in Fig. 9A,B. The IC_50_ of free DOX and DOX-loaded DOX-hyd-PEG-ALN micelle is showed in Table 2. The results indicated that there was no significantly difference in cytotoxicity between free DOX and DOX loaded DOX-hyd-PEG-ALN micelle when they were cultured with A549 cells for 24 h. However, after A549 cells were cultured with DOX loaded DOX-hyd-PEG-ALN micelle for 48 h, DOX loaded DOX-hyd-PEG-ALN micelle showed significantly higher anticancer activity at the dose of 40 μg/mL as compared with the same dose of free DOX. These results indicated that the DOX loaded DOX-hyd-PEG-ALN micelle was efficiently uptaken by A549 cells to produce the desired antitumor effect. The low anticancer activity of DOX loaded DOX-hyd-PEG-ALN micelle in 24 h was probably caused by the time-consuming DOX release from micelle and DOX trafficking from endolysosome to the nucleus in A549 cells.

### Cell cycle inhibition

**Figure 10:**
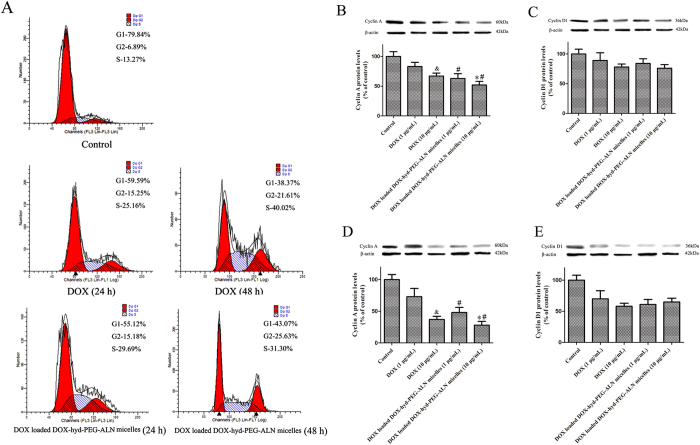
The effect of free DOX and DOX loaded DOX-hyd-PEG-ALN micelle on cell cycle of A549 cells. Representative flow cytometry pictures at the dose of 10 μg DOX/mL (**A**). The effect of free DOX and DOX loaded DOX-hyd-PEG-ALN micelle on the protein expression levels of cyclin A in 24 h (**B)** and 48 h (**D**). The effect of free DOX or DOX loaded DOX-hyd-PEG-ALN micelle on the protein expression levels of cyclin D1 in 24 h (**C)** and 48 h (**E**). Data are presented as the average ± standard deviation (n = 5). ^&^p < 0.05 vs 1 μg/mL free DOX; *p < 0.05 vs 1 μg/mL DOX loaded DOX-hyd-PEG-ALN micelle; ^#^p < 0.05 vs the same dose of free DOX.

DOX is reported to induce G_2_/M phase arrest^35^, thus the effect of DOX and DOX-loaded DOX-hyd-PEG-ALN micelle on cell cycle was analyzed by using flow cytometry. After A549 cells were treated with free DOX or DOX loaded DOX-hyd-PEG-ALN micelle for 24 h and 48 h, the quantitative analysis of the cell cycle distribution is showed in Table 3. Representative pictures are showed in Fig. 10A. Both free DOX and DOX loaded DOX-hyd-PEG-ALN micelle treated A549 cells displayed significant S and G_2_ phase arrest, and the extent of arrest augmented with the increase of incubation time. When free DOX and DOX loaded DOX-hyd-PEG-ALN micelle were respectively incubated with A549 cells for 24 h, the percentage of cells in G_2_ phase exhibited no significant difference between free DOX treated cells and DOX loaded DOX-hyd-PEG-ALN micelle treated cells. Compared with free DOX treated cells, the percentage of cells in S phase significantly increased in DOX loaded DOX-hyd-PEG-ALN micelle treated cells in 24 h. When DOX loaded DOX-hyd-PEG-ALN micelle and free DOX were respectively incubated with A549 cells for 48 h, the percentage of cells in G_2_ phase significantly increased both in DOX loaded DOX-hyd-PEG-ALN micelle treated cells and free DOX treated cells. Compared with free DOX, DOX loaded DOX-hyd-PEG-ALN micelles showed obviously higher cell cycle arrest capability in G_2_ phase in 48 h.

The cell cycle is regulated by several cyclins that drive cells in different phases. Cyclin A is involved in both S phase and G_2_/M transition of the cell cycle. Cyclin D1 is an important regulator of G1 to S phase progression^35^. Therefore, the effect of free DOX and DOX loaded DOX-hyd-PEG-ALN micelle on the protein expression levels of cyclin A and cyclin D1 was investigated. The results are showed in Fig. 10B–E. The results indicated that both free DOX and DOX loaded DOX-hyd-PEG-ALN micelle significantly decreased cyclin A protein levels in dose and time dependent manner. Compared with the same dose of free DOX, DOX loaded DOX-hyd-PEG-ALN micelle exhibited the stranger inhibition effect on cyclin A protein levels. This implied that DOX loaded DOX-hyd-PEG-ALN micelle had more cell cycle arrest capacity as compared with free DOX. On the other hand, both free DOX and DOX loaded DOX-hyd-PEG-ALN micelle significantly decreased cyclin D1 protein levels in time dependent manner. There was no significant difference in cyclin D1 protein levels between free DOX treated A549 cells and DOX loaded DOX-hyd-PEG-ALN micelle treated A549 cells.

### Drug biodistribution

**Figure 11:**
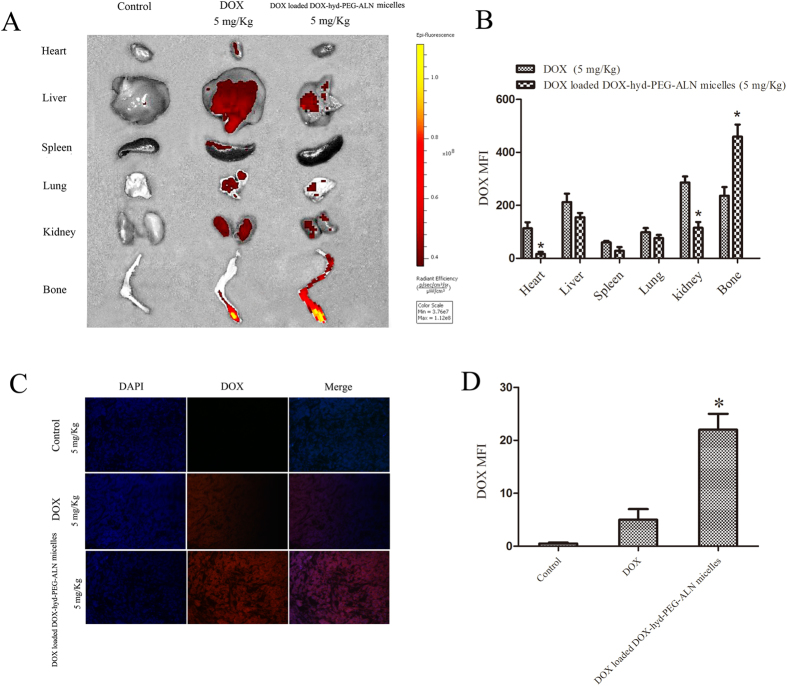
Tissue distribution of DOX detected by living image system (A) and distribution of DOX in the tumor tissue section detected by CLSM (C) at 24 h after free DOX (5 mg/kg) and DOX loaded DOX-hyd-PEG-ALN micelles (equivalent dose of DOX: 5 mg/kg) was intravenously administered to tumor-bearing nude mice. Quantitative analysis of in-Vivo Images (**B**, *P < 0.05 vs DOX in the same organ tissue) and CLSM images (**D**, *P < 0.05 vs DOX). Data are presented as the average ± standard deviation.

To further verify the active targeting capability of the DOX loaded DOX-hyd-PEG-ALN micelle, the distribution of DOX in different organs was observed and quantitatively analyzed by using the living image system. As showed in Fig. 11A,B, after free DOX was administered to tumor-bearing nude mice, red DOX fluorescence was distributed in heart, liver, lung and kidney tissue. There is a significant amount of red DOX fluorescence in leg bone tumor tissues after free DOX was intravenously administered. But in leg bone tumor tissues from DOX loaded DOX-hyd-PEG-ALN micelle treated tumor-bearing nude mice, the red DOX fluorescence intensity was approximately 2-fold higher than from free DOX treated tumor-bearing nude mice. After DOX loaded DOX-hyd-PEG-ALN micelle was intravenously administered, the red DOX fluorescence intensity was significantly reduced in heart and kidney as compared with free DOX treatment. The low concentration in tumor and high content in normal tissue led to the high systematic toxicity and low therapeutic index of free DOX. After DOX loaded DOX-hyd-PEG-ALN micelle was administered to tumor-bearing nude mice, a large amount of DOX was distributed in leg bone tumor tissue due to the EPR effect and the targeted moiety of the ALN. The high accumulation of DOX in leg bone tumor site could enhance its antitumor activity and decrease the systematic toxicity *in vivo*. Meanwhile, the red DOX fluorescence was also found in high blood perfused organs such as liver and lung after DOX loaded DOX-hyd-PEG-ALN micelle was intravenously administered. This was due to much blood passing through these organs and non specific uptake by MPS in these organs.

To observe whether DOX loaded DOX-hyd-PEG-ALN micelle could deliver DOX deeper in tumor tissue, the leg bone metastatic tumor tissue was sectioned and stained with DAPI, and red DOX fluorescence was quantitatively analyzed by using confocal laser scanning microscopy. As showed in Fig. 11C,D, in tumor tissue section from DOX-loaded DOX-hyd-PEG-ALN micelle treated mice, the red DOX fluorescence intensity was approximately 4-fold higher than from free DOX treated mice. In addition, DOX loaded DOX-hyd-PEG-ALN micelle treated tumor tissue section showed wide distribution of red DOX fluorescence throughout the whole tumor tissue section. This indicated that micelle could penetrate deeper in tumor tissue than free DOX.

In order to deliver enough amount of antitumor drug to tumor tissue, the drug loaded nanoparticle should be able to avoid the uptake by MPS. The absorption of plasma opsonin plays an important role in the process of macrophage recognition to nanoparticle. Much evidence indicated that nanoparticle with neutral or slight charge had lower opsonization rate than the same size nanoparticle with high charge^62^. Thus, macrophage uptake augments with the surface charge increasing, and the nanoparticles with the lowest absolute value of zeta potential can effectively decrease the uptake by MPS, thereby exhibiting a significant long blood circulation time and high accumulative rate in tumor tissue^60^. For example, Xiao K *et al.* found that compared with nanoparticle with zeta potential of −26.9 mV, nanoparticle with zeta potential of −17.5 mV exhibited higher uptake by tumor but lower uptake by liver and lung^51^. Furthermore, it has been found that the *in vivo* distribution and blood circulation time of nanoparticles are dependent on its size after being systemically administered. Small nanoparticles (10–20 nm) tend to widely distribute in various organs. Large particles (>1 μm) tend to aggregate under blood physiological conditions, be rapidly uptaken by MPS and accumulate mostly in the liver and spleen^60^. Kulkarni SA *et al.* reported that TPGS coated nanoparticle of 100 and 200 nm sizes could escape from MPS and thus prolong the half-life of the nanoparticle in the blood system. Small size (<50 nm) nanopartical usually accumulated in the liver and kidney^63^. The zeta potential and size of DOX loaded DOX-hyd-PEG-ALN micelle were −19.3 ± 4.5 mV and 114 ± 17 nm, respectively. The particle size and negative charged surface of DOX loaded DOX-hyd-PEG-ALN micelle conformed to the characteristics of long circulation time and high accumulation in tumor tissues of nanoparticle. Thus, the increased penetration and accumulation of DOX in tumor tissues were resulted from the suitable particle size and the negative charged surface of DOX loaded DOX-hyd-PEG-ALN micelle, which reduced non specific uptake by MPS and prolonged circulation time of micelle^49^,^64^,^65^.

### *In vivo* antitumor activity of DOX loaded DOX-hyd-PEG-FA micelle

**Figure 12:**
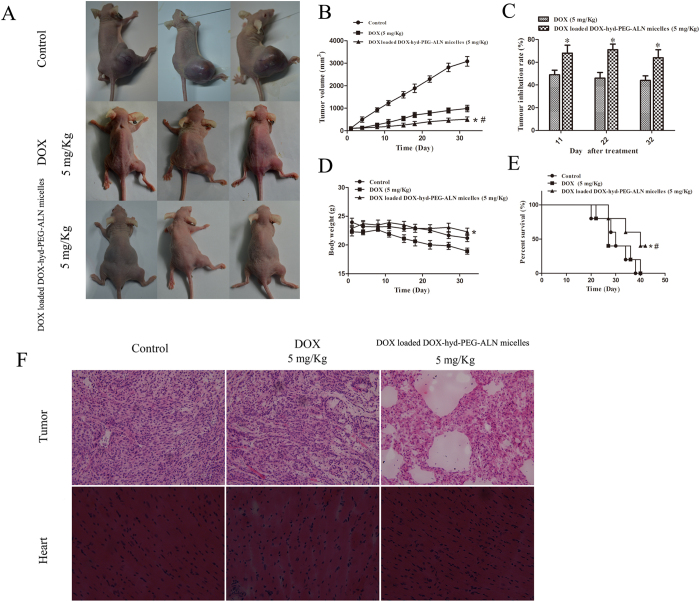
The *in vivo* antitumor activity of DOX loaded DOX-hyd-PEG-ALN micelles on tumor-bearing nude mice. Tumor-bearing nude mice recorded by camera at the end of treatment (**A**); tumor volume changes (**B**, *P < 0.05 vs DOX; ^#^P < 0.05 vs control); tumor inhibition rate (**C**, *P < 0.05 vs DOX at the same time point); body weight changes (**D**, *P < 0.05 vs DOX); survival curve (**E**, *P < 0.05 vs DOX; ^#^P < 0.05 vs control); H&E staining of tumor and heart tissue section from tumor bearing nude mice (**F**).

The *in vivo* antitumor experiment was performed in male nude mice bearing A549 cell tumor. As showed in Fig. 12A–C, free DOX exhibited obvious tumor growth inhibition *in vivo* as compared with control group. When the tumor-bearing nude mice were treated with DOX loaded DOX-hyd-PEG-ALN micelle, tumor growth was significantly delayed as compared with free DOX treated mice. The percentage survival curve is showed in Fig. 12E. The statistic analysis of survival of tumor-bearing nude mice is showed in Table 4. The results indicated that compared with free DOX treated tumor-bearing mice, the median survival is significantly longer in DOX loaded DOX-hyd-PEG-ALN micelle treated tumor-bearing nude mice.

Histological analysis of tumor tissue section was carried out to further evaluate the antitumor effect of DOX loaded DOX-hyd-PEG-ALN micelle, and the results are showed in Fig. 12F. Compared with tumor tissue section from normal saline and free DOX treated nude mice, tumor tissue section from DOX loaded DOX-hyd-PEG-ALN micelle treated mice showed serious vacuolation, inflammatory cell infiltration, and apoptosis. This result indicated that there were inflammatory and cytotoxic responses in tumor tissues when tumor-bearing nude mice were treated with DOX loaded DOX-hyd-PEG-ALN micelle.

**Figure 13:**
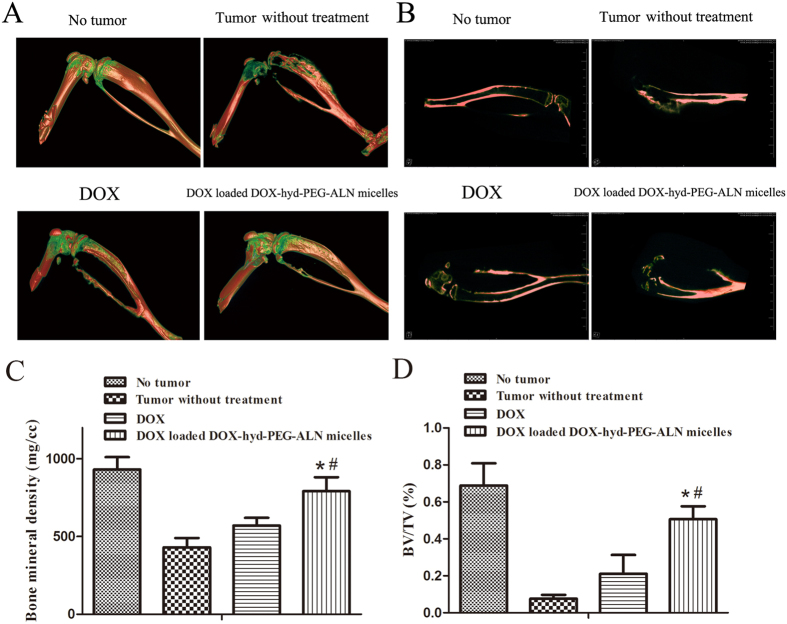
Micro-CT 3D-reconstruction image (A) and sagittal image (B) of leg bone of tumor-bearing nude mice. Micro-CT data quantitative analysis: (**C**) bone mineral density (BMD); (**D**) bone volume fraction (BV/TV). *P < 0.05 vs DOX; ^#^P < 0.05 vs tumor without treatment.

The therapeutic efficacy of DOX loaded DOX-hyd-PEG-ALN micelle on tumor-bearing nude mice was further evaluated by micro-CT, the results are showed in Fig. 13. The results indicated that tumor bone metastasis caused serious leg bone destruction and even leg bone fractures. After tumor-bearing nude mice were treated with DOX loaded DOX-hyd-PEG-ALN micelle, the leg bone mineral density (BMD) and the ratio between bone volume and tissue volume (BV/TV) in tumor site obviously increased as compared with free DOX treated tumor-bearing nude mice. This indicated that DOX loaded DOX-hyd-PEG-ALN micelle significant reduced bone destruction and decreased bone loss, both of which were caused by tumor bone metastasis. The superior therapeutic efficacy of DOX loaded DOX-hyd-PEG-ALN micelle was resulted from the high tumor tissue accumulation of DOX by the EPR effect and the high tumor cellular uptake efficiency of DOX loaded DOX-hyd-PEG-ALN micelle.

The side effect is one of the major concerns in the development of the novel drug delivery system. The change in body weight of the tumor-bearing nude mice is an important index to evaluate systemic adverse effects. As showed in Fig. 12D, the obvious body-weight loss was observed in free DOX and normal saline treated tumor-bearing nude mice, especially in the DOX treated tumor-bearing nude mice. Treatment with DOX loaded DOX-hyd-PEG-ALN micelle did not obviously cause body weight loss. The experiment results indicated that with the growth of tumor, tumor-bearing nude mice became emaciated. Thus, although tumor volume became enlarged as time prolonged in normal saline treated tumor-bearing nude mice, the body weight of tumor-bearing nude mice began falling slowly from the 20th day after tumor cells were inoculated. This result is consistent with what has already been reported^29^,^66^. The tumor-bearing nude mice treated with DOX loaded DOX-hyd-PEG-ALN micelle kept a vigorous and healthy appearance throughout the full experiment. Nevertheless, free DOX treated tumor-bearing nude mice showed weakened vitality. These observations indicated that the systemic side effects of DOX loaded DOX-hyd-PEG-ALN micelles were less than those of free DOX. Since DOX has exhibited serious dose-dependent toxicity to heart, the heart tissue was selected to evaluate the cardiotoxicity of DOX loaded DOX-hyd-PEG-ALN micelle. The results are showed in Fig. 12F. Compared with heart tissue section from normal saline treated nude mice, obvious neutrophils recruitment and cardiomyocyte hypertrophy were observed in heart tissue section that came from free DOX treated tumor-bearing nude mice. This indicated that free DOX showed evident cardiac toxicity. In contrast, no significant pathological changes were observed in heart tissue section that came from DOX loaded DOX-hyd-PEG-ALN micelle treated tumor-bearing nude mice. This is because DOX loaded DOX-hyd-PEG-ALN micelle delivered more DOX to tumor tissue but reduced the accumulation of DOX in heart. The above results suggested that DOX loaded DOX-hyd-PEG-ALN micelle could act as an ideal nano platform to deliver DOX into bone tumor tissues with less systemic toxicity.

## Conclusion

DOX loaded DOX-hyd-PEG-ALN micelle showed high drug loading, encapsulation efficiency, and binding affinity with HA. DOX loaded DOX-hyd-PEG-ALN micelle delivered more DOX into bone metastatic tumor tissues, which greatly improved the *in vivo* antitumor activity and reduced bone destruction with less systemic toxicity. DOX loaded DOX-hyd-PEG-ALN micelle had potential in the treatment of bone metastases cancer.

## Additional Information

**How to cite this article**: Ye, W.-l. *et al.* Doxorubicin-poly (ethylene glycol)-alendronate self-assembled micelles for targeted therapy of bone metastatic cancer. *Sci. Rep.* **5**, 14614; doi: 10.1038/srep14614 (2015).

## Figures and Tables

**Figure 1:**
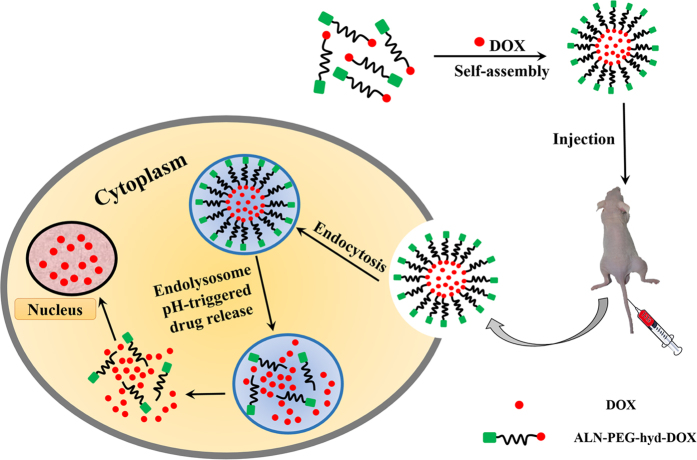
The schematic illustration of construction of DOX-loaded DOX-hyd-PEG-ALN micelle and pH triggered intracellular drug release. This figure was drawn by Wei-liang Ye. The mouse picture was taken by Wei-liang Ye during the animal experiment.

**Table 1:** The characterization of DOX loaded micelle.

DOX loaded micelle	Size (nm)	Zeta potential (mV)	PDI	DL (%)	EE (%)	CMC (mg/L)
DOX loaded DOX-hyd-PEG-ALN micelle	114 ± 17	−19.3 ± 4.5	0.142 ± 0.008	24.3 ± 3.7	64.3 ± 8.9	2.4 ± 0.6
DOX loaded DOX-hyd-PEG micelle	278 ± 20	−16.6 ± 3.8	0.265 ± 0.005	20.5 ± 2.5	55.6 ± 6.6	6.4 ± 1.7

DOX, doxorubicin; hyd, hydrazone; PEG, poly (ethylene glycol); ALN, alendronate; PDI, polydispersity index; DL, drug loading; EE, Encapsulation efficiency; CMC, critical micelle concentration. Data are mean ± SD (n = 3).

**Table 2:** Cytotoxicity of free DOX and DOX loaded DOX-hyd-PEG-ALN micelle on A549 cells.

Drugs	IC_50_(μg/mL)	
	24 h	48 h
Free DOX	3.4 ± 0.6	2.5 ± 0.6
DOX loaded DOX-hyd-PEG-ALN micelle	3.5 ± 0.4	1.3 ± 0.2*

^*^p < 0.05 vs DOX at the same time point.

**Table 3:** Effect of DOX and DOX loaded DOX-hyd-PEG-ALN micelle on A549 cell cycle.

Phase	Control	DOX		DOX loaded DOX-hyd-PEG-ALN micelle	
		24 h	48 h	24 h	48 h
G1	79.76 ± 0.37	59.67 ± 0.56	38.33 ± 0.98	55.37 ± 0.28	39.114 ± 0.55
G2	6.96 ± 0.16	15.18 ± 0.71	21.18 ± 1.35#	15.36 ± 0.52	26.86 ± 0.37*,#
S	13.36 ± 1.24	25.14 ± 1.46	40.15 ± 0.87#	29.92 ± 0.75*	32.72 ± 1.27#

^*^p < 0.05 vs DOX at the same time point.

^#^*P* < 0.05 vs 24 h. 10 μg DOX/mL.

**Table 4:** Survival statistic analysis of tumor-bearing nude mice.

Treatment group	Median survival (d)	Mean survival (d)	Maximal survival (d)	*P*
Control	30	29.13 ± 1.63	38	—
Free DOX	28	30.03 ± 1.68	40	0.376
DOX loaded DOX-hyd-PEG-ALN micelle	40	41.00 ± 1.31	42	0.013^a^

^a^compared with free DOX.
